# A repertoire of candidate effector proteins of the fungus *Ceratocystis cacaofunesta*

**DOI:** 10.1038/s41598-023-43117-7

**Published:** 2023-09-29

**Authors:** Gabriela N. Ramos-Lizardo, Jonathan J. Mucherino-Muñoz, Eric R. G. R. Aguiar, Carlos Priminho Pirovani, Ronan Xavier Corrêa

**Affiliations:** Departamento de Ciências Biológicas (DCB), Centro de Biotecnologia e Genética (CBG), Universidade Estadual de Santa Cruz (UESC), Ilhéus, BA 45662-900 Brazil

**Keywords:** Genomics, Protein function predictions, Computational biology and bioinformatics, Genetics

## Abstract

The genus *Ceratocystis* includes many phytopathogenic fungi that affect different plant species. One of these is *Ceratocystis cacaofunesta*, which is pathogenic to the cocoa tree and causes Ceratocystis wilt, a lethal disease for the crop. However, little is known about how this pathogen interacts with its host. The knowledge and identification of possible genes encoding effector proteins are essential to understanding this pathosystem. The present work aimed to predict genes that code effector proteins of *C. cacaofunesta* from a comparative analysis of the genomes of five *Ceratocystis* species available in databases. We performed a new genome annotation through an in-silico analysis. We analyzed the secretome and effectorome of *C. cacaofunesta* using the characteristics of the peptides, such as the presence of signal peptide for secretion, absence of transmembrane domain, and richness of cysteine residues. We identified 160 candidate effector proteins in the *C. cacaofunesta* proteome that could be classified as cytoplasmic (102) or apoplastic (58). Of the total number of candidate effector proteins, 146 were expressed, presenting an average of 206.56 transcripts per million. Our database was created using a robust bioinformatics strategy, followed by manual curation, generating information on pathogenicity-related genes involved in plant interactions, including CAZymes, hydrolases, lyases, and oxidoreductases. Comparing proteins already characterized as effectors in *Sordariomycetes* species revealed five groups of protein sequences homologous to *C. cacaofunesta.* These data provide a valuable resource for studying the infection mechanisms of these pathogens in their hosts.

## Introduction

*Ceratocystis* is one of many genera in the *Ceratocystidaceae* family (*Microascales, Sordariomycetes, Ascomycota*)^1^. The different species of this genus cause cankers and wilts in many economically important plant species^2^. For example, *Ceratocystis platani* causes severe wilt in plane trees (*Platanus*) in Europe, *Ceratocystis manginecans* produces mango tree wilt, and *Ceratocystis fimbriata *sensu stricto is a sweet potato pathogen^3^. *Ceratocystis cacaofunesta* is specific to cocoa trees and causes wilt associated with tree mortality in Central and South America^4^. Ceratocystis wilt of cacao was first described in 1918 in Ecuador and later found in Aragua state in Venezuela in the 1950s^5^. In Brazil, this pathogen was initially reported in the Amazon region. In the 1990s, *C. cacaofunesta* was introduced into the Southern Region of Bahia, one of the main cocoa-producing areas of Brazil^2^, where the disease has been responsible for losses of 20–30% in cocoa production^6^.

As a necrotrophic fungus, *C. cacaofunesta* causes cellular death during colonization and obstructs the transport of water and nutrients in plants, turning them yellow and then brown before they wither and die^7^. Its reproduction can occur asexually, through vegetative propagation and conidia formation, as well as sexually^8^. Plant infection mainly occurs through injuries, such as cuts, incurred by tools during agricultural practices and crop management, e.g., thinning and pruning, and by the attack of Coleoptera, e.g., *Xyleborus* sp. (*Coleoptera-Scolytidae*)^5^. After the onset of symptoms, the disease is difficult to control since the pathogen quickly and irreversibly infects and colonizes the vascular system^9^. Using fungicides, phytosanitary techniques, and selecting resistant cocoa tree varieties have been considered valuable strategies to control Ceratocystis wilt. However, it is difficult to control this disease due to the short period between the appearance of symptoms and the death of the plant^10^.

Phytopathogenic fungi mainly interact with their hosts through the secretion of protein effectors to promote pathogenesis^11^. These effectors are small molecules that help the pathogen successfully colonize the host plant and contribute to obtaining nutrients^12^. Criteria for defining candidate-secreted effector proteins (CSEP) include fungal proteins with a signal peptide for secretion, no transmembrane domain, small size, cysteine-rich, and mainly species-specific^13^. CSEPs can be classified according to their mode of release within the host. Thus, effectors are called extracellular if they are secreted into the apoplast or xylem of the host plant and cytoplasmic if they are translocated into host cells^14^. Advances in DNA sequencing techniques and high-throughput RNA sequencing (RNA-seq) technology coupled with falling costs have prompted the publication of many fungal genomes and led to the discovery of many new genes, making it possible to relate gene expressions to the physical symptoms of a disease^15,16^.

Analyzing these genomes allows the identification of effector proteins in different pathogenic fungi^17^. The in silico identification of these sequences is the first step on the road to functional characterization, which is very important to developing technology strategies to reduce losses caused by plant diseases^18^. In-silico analysis complemented by laboratory analysis allows the functional characterization of proteins related to pathogenicity, becoming key steps to identify colonization mechanisms in different host species^14^. The present study provides a database of protein effector candidates and identifies those most frequently expressed during the pathogen-host interaction in various species of *Ceratocystis*. These results allow us to understand different infection strategies of some *Ceratocystis* species and are a basis for additional laboratory studies on characterizing genes related to pathogenicity.

## Materials and methods

### Genome sequences

For this study, we selected the following five *Ceratocystis* genomes available in the National Center for Biotechnology Information’s (NCBI) GenBank. The genome of *C. albifundus* strain CMW17620, GCA_000813685.1, sequenced by the University of Pretoria^19^; the genome of *C. cacaofunesta* strain C1593, GCA_002776505.1, sequenced by Universidade Estadual de Campinas^8^; the genome of *C. fimbriata* strain CBS 114723, GCA_000389695.3, sequenced by the Forestry and Agricultural Biotechnology (FABI); the genome of *C. manginecans* strain CMW17570, GCA_000712455.1, also sequenced by the Forestry and Agricultural Biotechnology Institute^20^; and the genome of *C. platani* strain CFO, GCA_000978885.1, sequenced by the University of Neuchâtel in 2015 (Supplementary Table S1).

**Figure 1:**
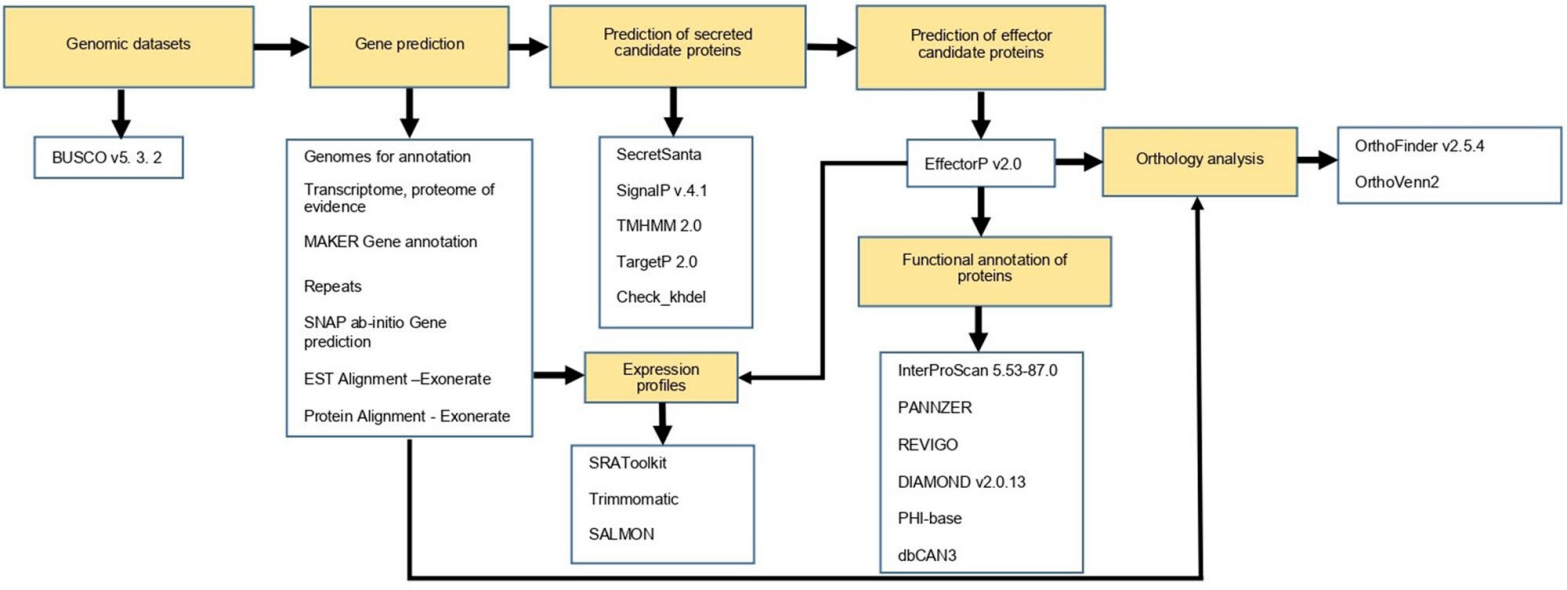
The workflow shows the steps for genome annotation and in-silico analysis of the secretome and effectorome of *Ceratocystis* species and the different bioinformatics tools used.

Different bioinformatics tools were used sequentially and complementarily in the multiple steps of in silico genomic analysis (Fig. 1). The quality and integrity of the genomes were evaluated using Benchmarking Universal Single-Copy Orthologs version 5. 3. 2 (BUSCO v5. 3. 2.). For this purpose, BUSCO was configured using the *Sordariomycetes* lineage^21^.

### Gene prediction

Although a gene prediction of the genome of *C. cacaofunesta* is available via the NCBI^2^, even so, a new gene prediction was made using updated sequences-evidences in the homology prediction stages and updated ab initio models. This way, it standardized all the genomes used in the present study, along with the species *C. albifundus* and *C. manginecans*, which still lacked gene prediction. Genetic prediction of the genome of these three species was predicted using the MAKER 3.01.04 annotation pipeline^22^. A combination of ab initio and homology-based methods (transcriptome data and homology with known proteomes) was used for gene prediction. Before gene prediction, all sequences were masked with RepeatMasker^23^. For the ab initio prediction, two iterations were performed using SNAP through the Hidden Markov Model (HMM)^24,25^. For homology-based gene prediction, Exonerate^26^ was used with the est2genome and protein2genome alignment methods; for the est2genome method, evidence transcriptomes of the *C. platani* GCA_000978885.1 and *C. fimbriata* GCA_000389695.3 were used. For the protein2genome method, the known protein sequences of each species were used: the reference proteome identified with ID: UP000034841 was used for the species *C. platani*, and the proteome with the ID: UP000222788 used for *C. fimbriata*, both available on the Uniprot database (Supplementary Table S1).

### Secretome and effectorome prediction

In silico predictions of secreted proteins were characterized using the SecretSanta package through the R interface; the input files provided were the proteomes resulting from the gene prediction of the species *C. albifundus*, *C. cacaofunesta, C. manginecans*, and the reference proteomes of the species *C. platani* and *C. fimbriata*^27,28^. Secretome prediction requires multiple steps. One of these steps is the identification of the signal peptide (SP), which was carried out through SignalP v.4.1 software^29^. In the second step, we use TMHMM 2.0 software to identify sequences without transmembrane (TM) domains, providing as input file the result obtained in step one^30^. Subsequently, extracellular localization prediction was performed using TargetP 2.0 software. TargetP 2.0 output sequences were compiled to search for endoplasmic reticulum (ER) retention signals using Check_khdel software^27^. A manual curation of the sequences resulting from the secretome was performed, and those that were cut in any step of SecretSanta were recovered to obtain the complete sequences. Finally, to distinguish between cytoplasmic and apoplastic effectors, EffectorP v2.0 software was used, considering as candidate proteins those peptides with a minimum size of 30 amino acids and a maximum of 600 amino acids^31^.

### Expression profiles

Transcriptome data of *C. cacaofunesta* and *C. fimbriata* species were downloaded from the SRA databank (https://www.ncbi.nlm.nih.gov/sra) and converted into FASTQ format using SRAToolkit. The Trimmomatic^32^ tool was used to remove sequence adapters and low-quality reads using the PHRED index quality score. The gene expression of the gene coding for candidate effector proteins was quantified using the SALMON^33^ software. First, the transcriptomes of *C. fimbriata* (GCA_000389695.3) and *C. cacaofunesta* (Supplementary Table S2) were used to create an index of the transcripts using the SALMON-index. Then, the transcript index resulting from the SALMON-index and the available RNASeq reads from *C. cacaofunesta* (SRR6217952) and *C. fimbriata* (SRR8599076) were used to obtain a quantification using SALMON-quant. The Salmon result was filtered to get the number of transcripts per million (TPM) of those transcripts coding for candidate effector proteins that were the result of EffectorP software.

### Functional annotation

Functional characterization of candidate effector proteins was performed using InterProScan 5.53–87.0^34^ and PANNZER^35,36^. To analyze the resulting GO terms, we use the REVIGO^37^ software with the simRel parameters, which provides a measure of functional similarity to compare two GO terms. For p-values, terms with the highest “singularity”, average, or negative similarity of a term to all other terms were prioritized using a cut-off value of 0.7. The comparison was made against the entire database of UniProt^38^. To identify cerato-platanin proteins (CPPs), a sequence alignment was carried out with the proteome of the five *Ceratocystis* species through DIAMOND v2.0.13^39^ using the CPP sequence (ID: KKF93197.1) available from NCBI.

The identification and classification of CAZymes related to the candidate effector proteins of the five *Ceratocystis* species was performed through the dbCAN3 server using the candidate effector protein sequences as input files with default configuration parameters and E-value < 1e−15, coverage > 0.35^40^. BLASTp analysis of the candidate effector proteins from the five *Ceratocystis* species was performed against the Pathogen Host Interaction PHI-base V.4.14 database (http://www.phi-base.org) with identity parameters > 25, E-value: 1.0e−5^41^. To obtain those genes related to pathogenicity and that had a higher level of expression, the PHI-base result of the *C. cacaofunesta* and *C. fimbriata* species was filtered by selecting 20 candidate effector proteins that presented the highest number of TPM. These 20 chosen proteins were divided into ten apoplastic effector candidates with the highest TPM and ten cytoplasmic effector candidates with the highest TPM for each species.

### Analysis of orthologous gene families in *Sordariomycetes*

An orthology analysis was performed using OrthoFinder v2.5.4^42^ and OrthoVenn^43^ software. For the orthology analysis performed with OrthoFinder v2.5.4, the UniProt reference proteomes of *C. platani* (ID: UP000034841) and *C. fimbriata* (ID; UP000222788) were used, as well as the proteomes resulting from the genetic prediction of *C. cacaofunesta, C. manginecans,* and *C. albifundus*. Additionally, the proteomes of five related *Sordariomycetes* species were used: *Verticillium dahliae* (ID: UP000001611), *Verticillium alboatrum* (ID: UP000008698), *Fusarium oxysporum* strain Fo5176 (ID: UP000002489), *Fusarium verticillioides* 7600 (ID: UP000009096), and *Fusarium graminearum* PH-1 (ID: UP000070720) (Supplementary Table S1). For orthology comparison using OrthoVenn, sequence similarity parameters with a cut-off value of 1e−2 and an inflation value of 1.5 were used, and the sequences considered as candidate effector proteins resulting from the analysis carried out with the EffectorP software for each of the five *Ceratocystis* species were used as input files.

## Results

Five *Ceratocystis* species were selected, considering the availability of the assembled genome and assessment of the genome assembly quality metric characteristics (Table 1). In the case of selected species with more than one assembled genome, only one of these was chosen according to the level of integrity that the genome presented, always prioritizing the most complete available. Those selected were *C. albifundus* GCA_000813685.1, *C. cacaofunesta* GCA_002776505.1, *C. fimbriata* GCA_000389695.3, and *C. manginecans* GCA_000712455.1, assembled at scaffold level, as well as *C. platani* GCA_000978885.1, assembled at contigs level.

**Table 1:** Comparative analysis of metrics referring to the genome and proteome of a lineage of each of the following species: *Ceratocystis albifundus, C. cacaofunesta, C. fimbriata, C. manginecans,* and *C. platani*.

Species	*C. albifundus*	*C. cacaofunesta*	*C. fimbriata*	*C. manginecans*	*C. platani*
Strain	CMW17620	C1593	CBS114723	CMW17570	CFO
Assembled genome size (Mb)	26.88	30.48	30.16	31.71	29.18
Number of scaffolds	1405	603	399	980	1213
N50	20,627	54,222	23,089	30,399	77,580
Number of contigs	2894	1442	2524	2295	1213
GC content (%)	48.6	48.1	47.6	47.9	48.2
Predicted proteome (number of sequences)	7619	7879	7266	7563	5622

The BUSCO software performed quality analysis for each genome (Table 2). In this analysis, it was possible to observe the genomic completeness of 94.3% for *C. cacaofunesta,* 94.3% for *C. fimbriata*, 94.1% for *C. manginecans*, 93.7% for *C. albifundus*, and 94.2% for *C. platani.* The gene prediction of the genomes performed through the MAKER2 pipeline allowed us to predict 7879, 7619, and 7563 proteins for *C. cacaofunesta, C. albifundus*, and *C. manginecans*, respectively (Table 1).

**Table 2:** Quality analysis of *Ceratocystis* sp*.* genomes; *C. albifundus, C. cacaofunesta, C. fimbriata, C. manginecans,* and *C. platani*, results of the BUSCO categories (complete (C), complete and single copy (S), complete and duplicated (D), fragmented (F), missing (M), n: gene number).

Species	BUSCO notation assessment result
*C. albifundus*	C: 93.7% [S: 93.6%, D: 0.1%], F: 0.4%, M: 5.9%, n: 3817
*C. cacaofunesta*	C: 94.3% [S: 94.2%, D: 0.1%], F: 0.3%, M: 5.4%, n: 3817
*C. fimbriata*	C: 94.3% [S: 94.1%, D: 0.2%], F: 0.2%, M: 5.5%, n: 3817
*C. manginecans*	C: 94.1% [S: 93.9%, D: 0.2%], F: 0.4%, M: 5.5%, n: 3817
*C. platani*	C: 94.2% [S: 94.0%, D: 0.2%], F: 0.3%, M: 5.5%, n: 3817

### Secretome and effectorome

**Figure 2:**
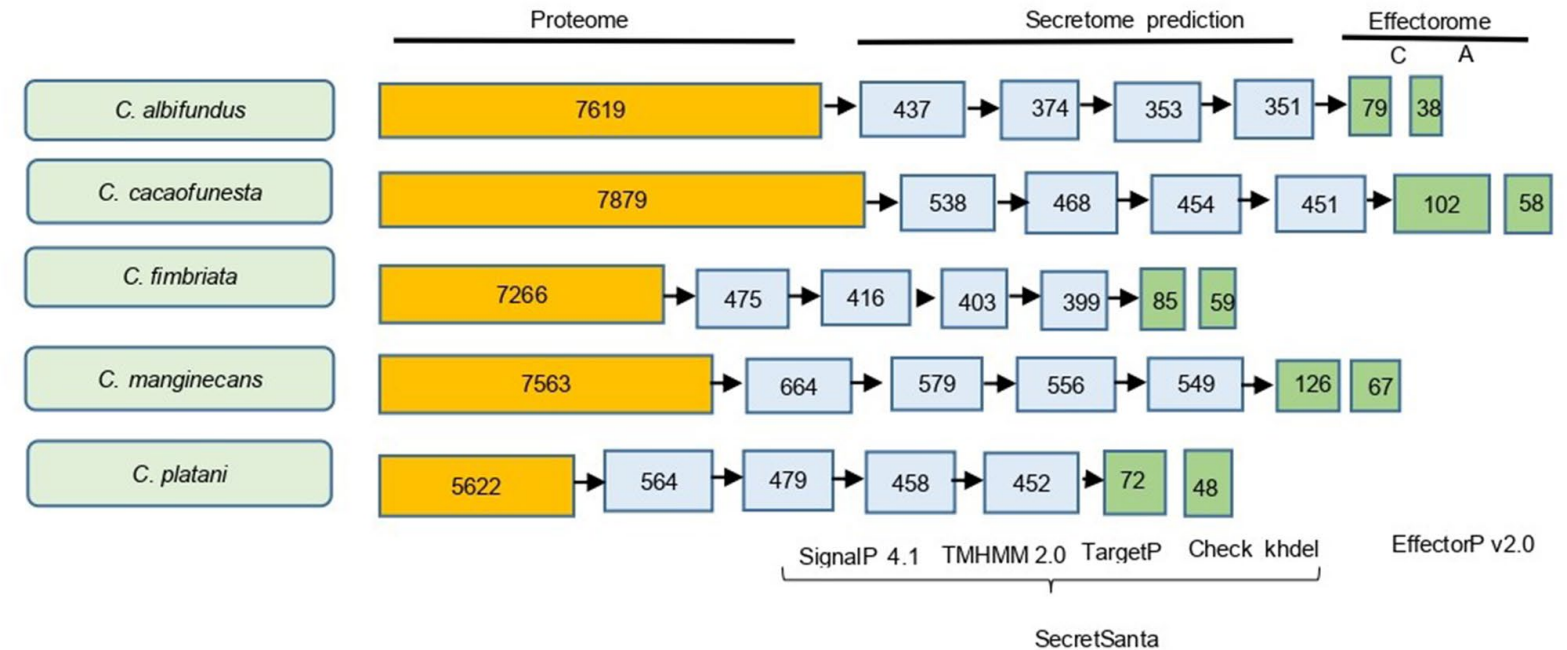
Pipeline for in silico characterization of secreted effector proteins. The figure shows the hypothetic proteome, secretome, and effectorome of *Ceratocystis* sp. The first column contains the *Ceratocystis* species names. Column two shows the amount sequences of the proteome of each species. Column three is the number of sequences that have a signal peptide. Column four indicates the number of sequences with a signal peptide and without transmembrane domains. Column five shows the number of sequences with a signal peptide, without transmembrane domains, and with extracellular localization. Column six shows the number of sequences with a signal peptide, without transmembrane domains, with extracellular localization and no ER retention signals, and the total number of sequences belonging to the secretome of the different species. The columns in green show the number of sequences of effector candidate proteins: column seven represents effector candidate proteins with possible direction to the cytoplasm (C), and column eight those directed to the apoplast (A).

The secretome of each *Ceratocystis* species object of this study was predicted from the proteome using the SecretSanta pipeline, which uses a series of combined software to predict and classify the secreted proteins. For this purpose, all 7619 proteins from *C. albifundus*, 7879 from *C. cacaofunesta*, 7266 from *C. fimbriata,* 7563 from *C. manginecans,* and 5622 from *C. platani*, were examined (Supplementary Table S3). Those that fit into all of the following four categories were considered to be secreted proteins: I, proteins containing signal peptides; II, proteins containing a signal peptide and lacking a transmembrane domain; III, proteins containing a signal peptide without a transmembrane domain and with extracellular localization; and IV, secreted proteins containing a signal peptide without a transmembrane domain, with extracellular localization and without ER retention signal (Fig. 2).

In total, our Bioinformatics strategy predicted 399 secreted proteins for *C. fimbriata*, 452 for *C. platani*, 451 for *C. cacaofunesta*, 351 for *C. albifundus* and 549 for *C. manginecans*, representing 5.49%, 8.04%, 5.72%, 4.61% and 7.26% of all predicted proteins, respectively (Supplementary Table S4). The candidate effector proteins were identified using EffectorP for each of the species were: a total of 144 candidate effectors belonging to *C. fimbriata*, consisting of 85 cytoplasmic and 59 apoplastic candidate effectors; 120 candidate effectors for *C. platani*, 72 cytoplasmic and 48 apoplastic; 160 candidate effectors for *C. cacaofunesta*, with 102 cytoplasmic and 58 apoplastic candidate effectors; 117 for *C. albifundus*, of which 79 were predicted as cytoplasmic candidate effectors and 38 as apoplastic candidate effectors, and 193 for *C. manginecans*, with 126 cytoplasmic and 67 apoplastic candidate effectors (Fig. 2, Supplementary Table S5).

### Expression profiles

The expression profile of *C. cacaofunesta* and *C. fimbriata* effector candidate proteins showed that of the 160 *C. cacaofunesta* candidate effector proteins, 146 showed expression values, the apoplastic effector candidate proteins of *C. cacaofunesta* showed an average of 304.627 TPM, and the cytoplasmic effector candidate proteins showed an average of 108.46 TPM and only 14 did not show values (Supplementary Table S6). For *C. fimbriata,* 128 candidate effector proteins showed non-zero TPM values, 54 candidate effector proteins directed to the apoplastic with an average of 283.395 TPM, and 74 candidate cytoplasmic effector proteins with an average of 104.956 TPM (Supplementary Table S6).

### Functional annotation and classification of effector proteins

**Figure 3:**
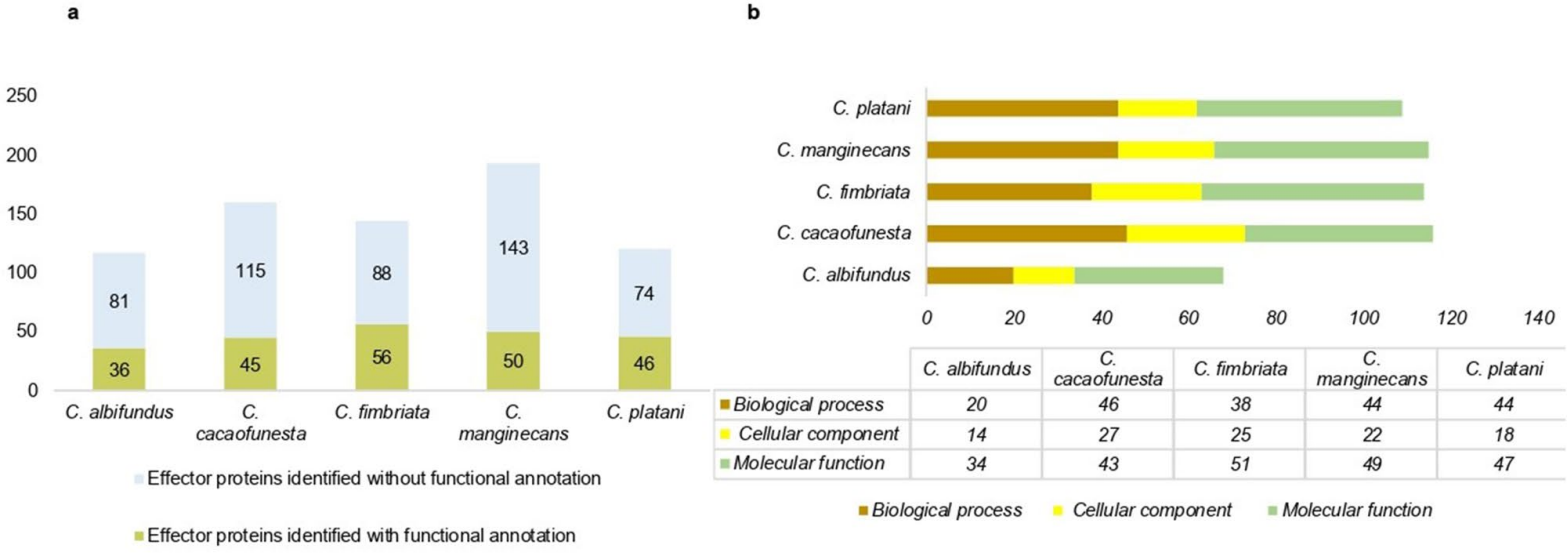
(**a**) The bar graph shows in green color the total number of candidate effector proteins identified with functional annotation in each Ceratocystis species and in blue color the total number of candidate effector proteins that do not have functional annotation. (**b**) The bar graph shows the total number of GO terms of each Ceratocystis species, divided according to the biological processes, molecular function, and cellular component in which they are involved.

Candidate effector proteins identified with functional annotation were separated according to the biological processes, molecular function, and cellular components in which they are involved. Only some of the total number of candidate effector proteins predicted in the different species had known functions: 56 proteins for *C. platani*, 50 for *C. manginecans,* 46 for *C. fimbriata*, 45 for *C. cacaofunesta* and 36 for *C. albifundus* (Fig. 3a).

In the GO level biological process, 20 GO terms were related to *C. albifundus*, 46 to *C. cacaofunesta,* 38 to *C. fimbriata*, 44 to *C. manginecans*, and 44 to *C. platani* (Supplementary Table S7). Regarding the GO level related to molecular functions, 34 belonged to *C. albifundus*, 43 to *C. cacaofunesta*, 51 to *C. fimbriata*, 49 to *C. manginecans,* and 47 to *C. platani* (Supplementary Table S8). And for the level of cellular components, 14 were related to *C. albifundus,* 27 to *C. cacaofunesta*, 25 to *C. fimbriata*, 22 to *C. manginecans,* and 18 to *C. platani* (Fig. 3b, Supplementary Table S9).

Of the GO terms found, 42 were common to the five *Ceratocystis* species classified into three different categories: 22 GO terms belong to the category of molecular function, being hydrolase activity the most represented; 13 GO terms in the category of biological processes represented by carbohydrate metabolic process; and seven GO terms in the category of cellular components, the most representative being the integral component of membrane (Table 3).

**Table 3:** Top 10 significantly enriched GO terms for common candidate effector proteins in all five *Ceratocystis* species.

GO term	GO-ID	Category	Log size
Carbohydrate metabolic process	GO:0005975	Biological process	6.23
Protein folding	GO:0006457	Biological process	5.48
Proteolysis	GO:0006508	Biological process	6.17
Lipid metabolic process	GO:0006629	Biological process	6.11
Integral component of membrane	GO:0016021	Cellular component	7.13
Protein binding	GO:0005515	Molecular function	6.23
ATP binding	GO:0005524	Molecular function	6.67
Zinc ion binding	GO:0008270	Molecular function	6.10
Hydrolase activity	GO:0016787	Molecular function	6.84
Catalytic activity, acting on a protein	GO:0140096	Molecular function	6.58

**Figure 4:**
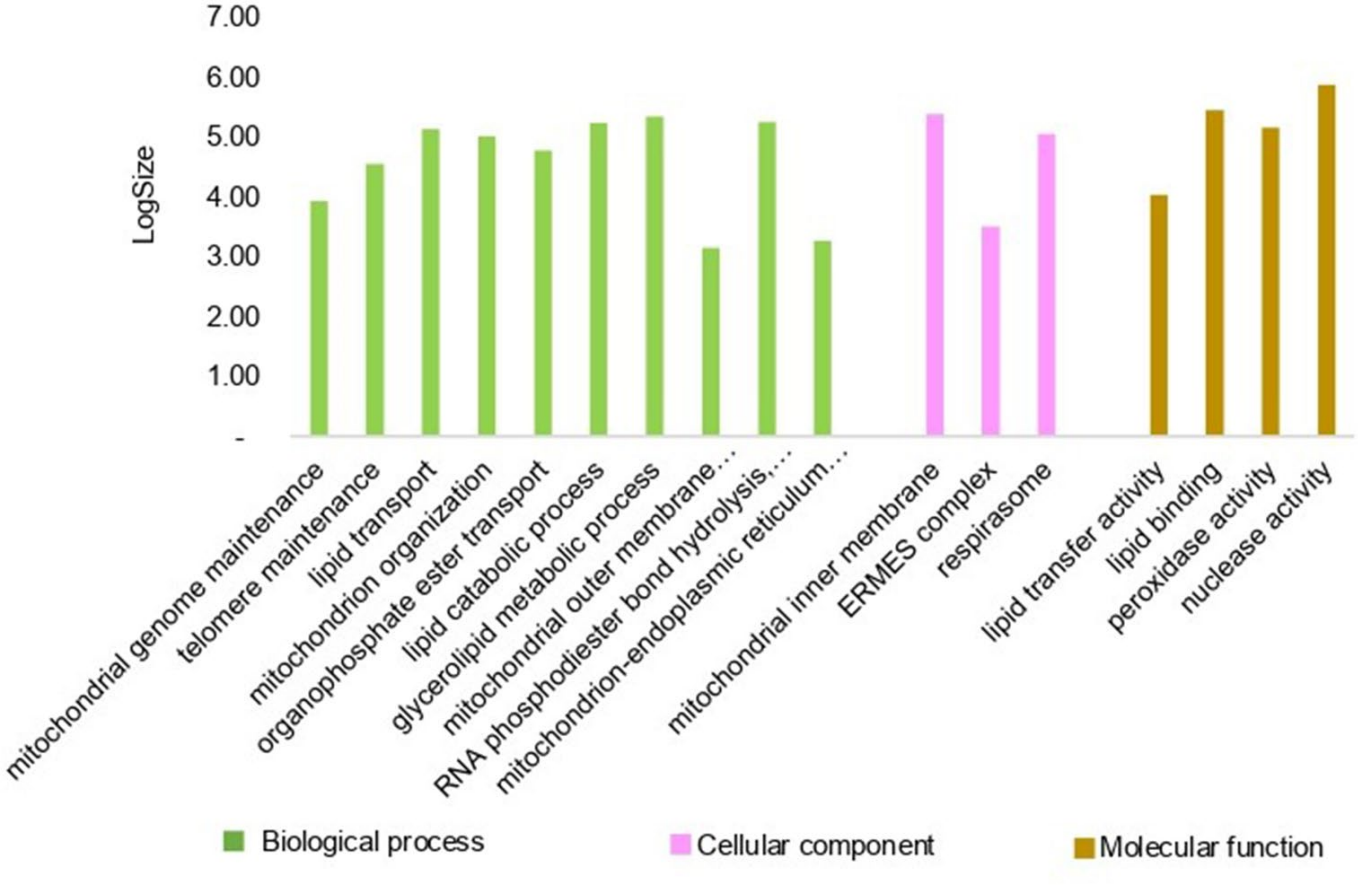
The bar chart shows GO terms unique to *C. cacaofunesta*, GO terms related to biological processes in green, GO terms associated with the cellular component in pink, and related to molecular function in brown.

Other GO terms were exclusive for each species. So, *C. cacaofunesta* shows 19 exclusive GO terms distributed in the three categories (Fig. 4). The species *C. fimbriata* and *C. albifundus* presented 12 exclusive GO terms per species. The most represented for *C. fimbriata* are related to the biosynthetic process of carbohydrate derivatives and the metabolic process of cellular proteins, and for *C. albifundus,* related to the metabolic processes of organic substances and nitrogenous compounds. *C. manginecans* presented six exclusive GO terms represented by rRNA processing and the RNA catabolic process. For the species *C. platani* four exclusive GO terms are related to inserting a tail-anchored membrane protein into the ER membrane and carbon lyase activity (Supplementary Tables S7, S8, and S9).

The alignment of the cerato-platanin sequences (CPPs) against each of the proteomes of the five *Ceratocystis* species showed 88% sequence similarity for each species. When performing a functional annotation through PHI-base, it was observed that the category with the highest percentage of identity was related to hypervirulence, directly related to the increase in virulence (Supplementary Table S10).

The annotations made through dbCAN for the identification of CAZymes, showed a total of 62 CAZymes functionally classified into five categories: glycosyltransferases (GTs), glycoside hydrolases (GHs), polysaccharide lyases (PLs), carbohydrate esterase (CEs), and auxiliary activities (AAs). The most abundant in the five species of *Ceratocystis* was the GHs with six (GH16_1, GH16_10, GH5_5, GH132, GH7, GH43_6) in the species *C. albifundus,* eight (GH18, GH43_6, GH28, GH11, GH16_1, GH7, GH132, GH16_19) in *C. cacaofunesta,* eight (GH11, GH43_6, GH7, GH16_1, GH132, GH7, GH28, GH16_1) in *C. fimbriata*, six (GH132, GH28, GH11, GH7, GH16_19, GH18) in *C. manginecans*, and five (GH43_6, GH16_19, GH11, GH132, GH28) in *C. platani* (Supplementary Table S11).

The second most abundant category is the PLs, with two (PL1_9, PL1_4) for *C. albifundus*, two (PL1_4, PL3_2) for *C. cacaofunesta*, four (PL1_9, PL1_4, PL3_2, PL1_4) for *C. fimbriata*, three (PL1_4, PL3_2, PL1_9) for *C. manginecans,* and three (PL3_2, PL1_9, PL1_4) for *C. platani.* We have the following trends for other categories: the AAs category showed one (AA11) for each species *C. albifundus, C. cacaofunesta, C. manginecans*, and two for *C. platani.* The GTs category showed one (GT32) for each species *C. cacaofunesta, C. fimbriata, C. manginecans*, and two (GT34, GT32) for *C. platani.* Finally, in the CEs category, there was one (CE1) for each species *C. fimbriata, C. manginecans*, and *C. platani* (Supplementary Table S11).

**Figure 5:**
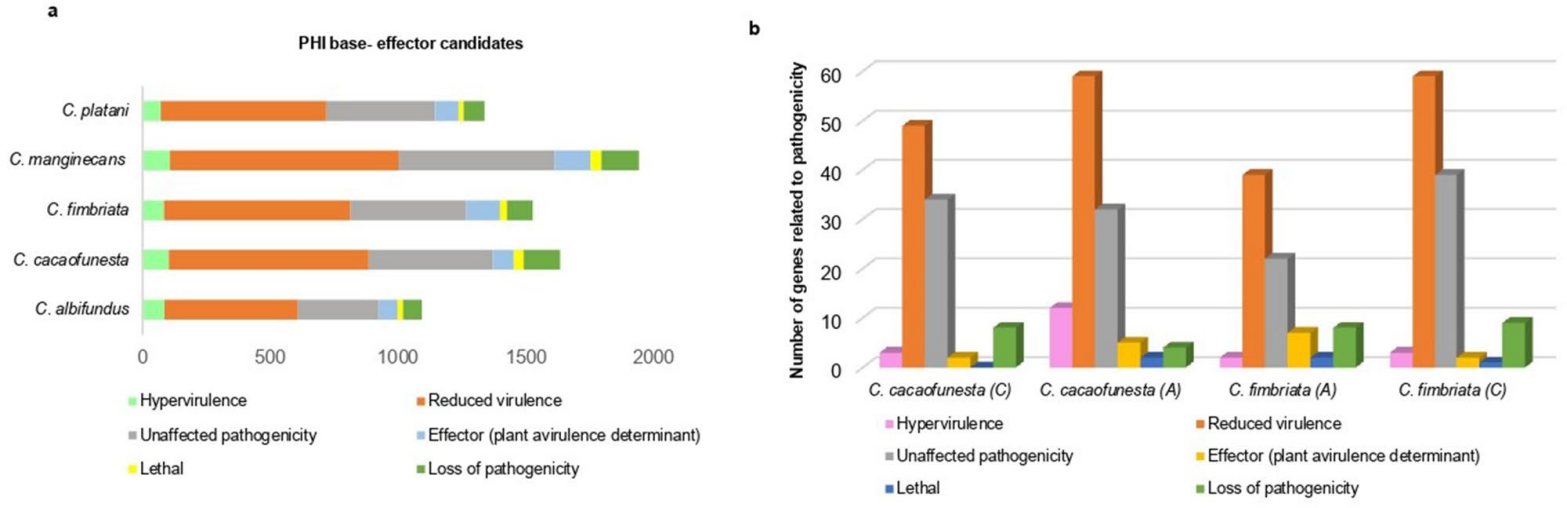
Genes of *Ceratocystis* species involved in pathogenicity. (**a**) Total potential pathogenic genes classified in the different PHI-base categories for each of the five *Ceratocystis* species. (**b**) Number of genes related to pathogenicity in 20 candidate effector proteins with the highest TPM for the species *C. cacaofunesta* and *C. fimbriata*, the letter (A) represents apoplastic and the letter (C) cytoplasmic.

Of the total number of candidate effector proteins for each of the five *Ceratocystis* species annotated with PHI-base, all the proteins obtained at least one hit in the annotation, showing a total of 7541 genes divided among five species, with a higher number of genes in the species: *C. manginecans* (1943), *C. cacaofunesta* (1636), and *C. fimbriata* (1529). The 1943 genes found in the species *C. manginecans* were classified into different categories, showing a greater amount in the category reduced virulence (897) and unaffected pathogenicity (610), followed by proteins related to loss of pathogenicity (146), and others identified as an effector (plant avirulence determinant) (141), and those about hypervirulence (107) and proteins lethal (42) (Fig. 5a, Supplementary Table S12).

For the 1636 sequences of *C. cacaofunesta*, the most significant values were for the reduced virulence category (783) and unaffected pathogenicity (486), followed by proteins identified with loss of pathogenicity (144), hypervirulence (103), effector (plant avirulence determinant) (83), and proteins lethal (37). And for *C. fimbriata*, the most represented categories are related to reduced virulence (732) and unaffected pathogenicity (453), followed by an effector (plant avirulence determinant) (131), loss of pathogenicity (102) and those related to hypervirulence (83), and proteins lethal (28) (Fig. 5a, Supplementary Table S12).

The species *C. platani* showed a total of 1339 genes, with the most represented category of reduced virulence (648) and unaffected pathogenicity (427), followed by proteins identified as effector (plant avirulence determinant) (92) and others related to hypervirulence (86), loss of pathogenicity (71) and protein lethal (19). And the species with the least number of genes was *C. albifundus*, with a total of 1094 genes, where the majority were related to reduced virulence (522) and unaffected pathogenicity (317), followed by proteins identified with hypervirulence (86), loss of pathogenicity (74), effector (plant avirulence determinant) (74), and proteins lethal (21) (Fig. 5a, Supplementary Table S12).

The 20 candidate effector proteins that showed the highest TPM in *C. cacaofunesta* presented similarity with 210 genes related to pathogenicity, offering the most representative levels in the reduced virulence category (59 genes encoding candidate apoplastic effector proteins and 49 genes encoding cytoplasmic ones). In the unaffected pathogenicity category, we observed 32 apoplastic genes and 34 cytoplasmic genes; for the hypervirulence category, 12 apoplastic genes and three cytoplasmic genes. The categories with the lowest numbers of genes were the effector (plant avirulence determining) with five apoplastic genes and two cytoplasmic genes, the loss of pathogenicity category with four apoplastic and eight cytoplasmic genes, and finally, the lethal genes category with only two apoplastic effectors genes (Fig. 5b, Supplementary Table S13).

For the *C. fimbriata*, the 20 candidate effector proteins with the highest number of TPMs presented similarity with 193 genes related to pathogenicity, showing the most representative levels in the reduced virulence categories, with 39 genes encoding candidate apoplastic effector proteins and 59 genes encoding apoplastic ones. Then comes the unaffected pathogenicity category, presenting 22 apoplastic genes and 39 cytoplasmic genes. The categories that have a lower number of genes were related to loss of pathogenicity with eight apoplastic genes and nine cytoplasmic genes; the effectors (plant virulence determinant) category with seven apoplastic genes and two cytoplasmic genes; the genes related to hypervirulence being two apoplastic genes and three cytoplasmic genes; and in the lethal genes category with two apoplastic genes and three cytoplasmic genes (Fig. 5b, Supplementary Table S13).

### Orthology analysis

**Figure 6:**
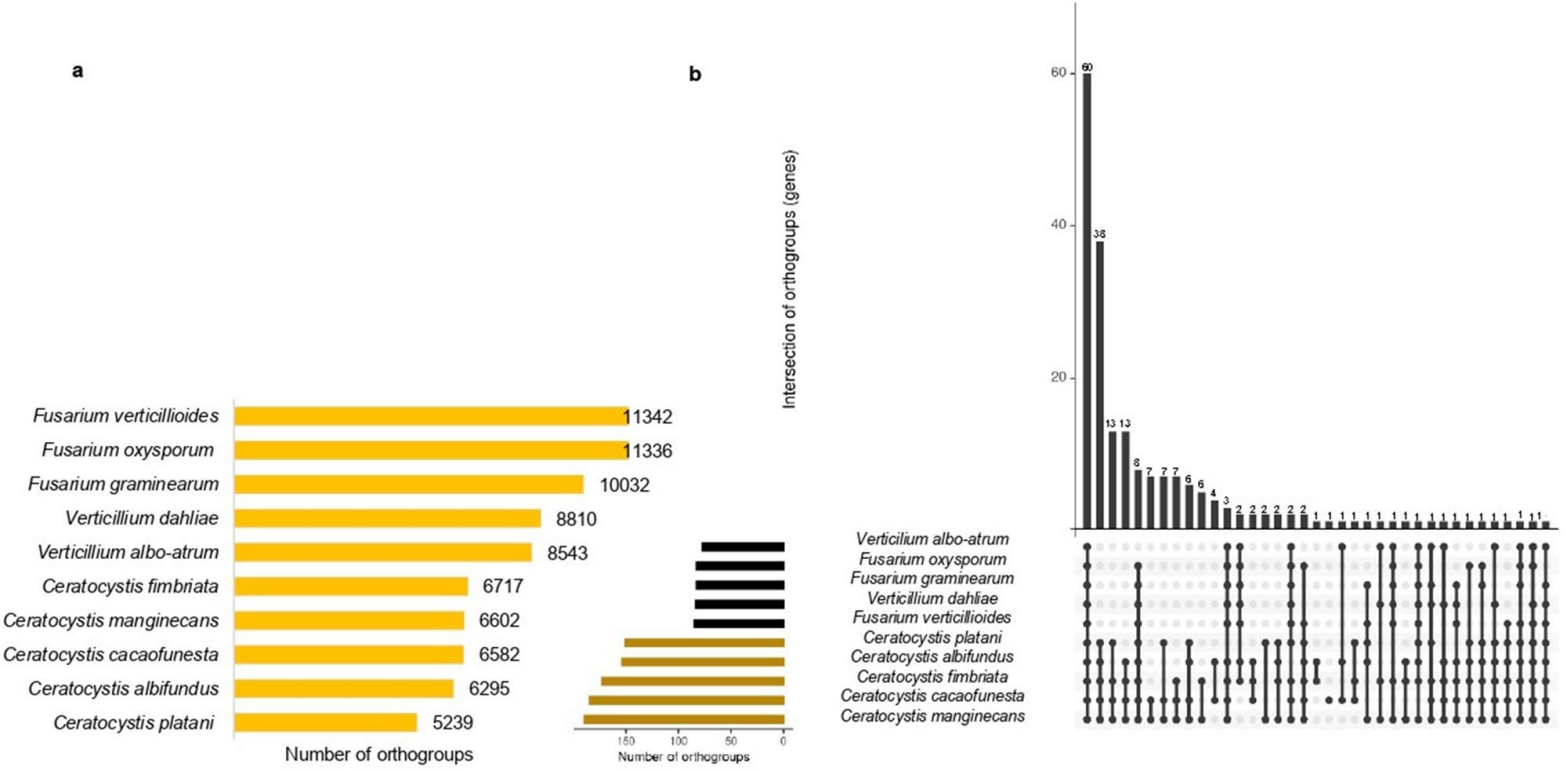
Groups of orthologous genes of ten *Sordariomycetes* species from orthoFinder analysis. (**a**) *Sordariomycetes* fungus species compared in this analysis, the graphic shows the species name and the total number of orthogroups in each proteome. (**b**) Comparative genomic analysis of five target species of *Ceratocystis* and five other species of *Sordariomycetes*. The Upset plot of the protein cluster analysis shows in the bars on the upper side the number of orthogroups shared by the species highlighted in the black dots on the lower side.

OrthoFinder analysis of the different selected *Sordariomycetes* fungi enabled the identification of groups of orthologous genes among the proteins of the ten species of *Sordariomycetes.* The proteomes of these ten species were assigned to a total of 14,605 orthogroups, with the *Ceratocystis* species providing a smaller amount than the other proteomes (Fig. 6a). Comparison of the orthology of *Ceratocystis* candidate effector proteins with the other *Sordariomycetes* species showed that only 207 of the total number of orthogroups found encode candidate effector proteins within the five species of *Ceratocystis*; out of these, 60 orthogroups were common to all ten species of *Sordariomycetes*, with a total of 950 effector proteins. Only four of the 60 orthogroups showed functional annotation against the Swiss-Prot database (Fig. 6b).

The four orthogroups shared among the ten species show functional annotation with the following proteins: the Effector Vd424Y (ID: G2X4G0), secreted by *V. dahliae* located in the host nucleus and contributes to the virulence process. Four proteins with cellular component function secreted by the fungus *F. graminearum*, one of them acting in the extracellular region and in the host cell nucleus, causing induction of cell death and hydrogen peroxide accumulation in wheat leaves known as endo-1,4-beta-xylanase B protein (ID: I1RII8); another known effector protein acting on the Golgi membrane, cytoplasmic vesicle membrane, and mitochondrial membrane, related to biological processes such as protein transport, identified as Autophagy-related protein 27 (ID:I1RD82). The Endoplasmic reticulum vesicle protein 25 (ID: Q4HY20) functions in the Golgi membrane, endoplasmic reticulum membrane, and integral membrane component, performing biological processes of protein transport and vesicle-mediated transport. The Aurofusarin biosynthesis cluster protein S (ID: I1RF63) mediates the biosynthesis of Aurofusarin, a red pigment of the mycelium that acts as a mycotoxin (Supplementary Tables S14, S18).

Another eight orthogroups with 84 proteins were found in all species except for *V. alboatrum*. Likewise, three orthogroups (28 proteins) were found in most species, except *C. platani*; one of them showed functional annotation related to the RNA exonuclease four protein (ID: Q4IEV5) of *F. graminearum* with cellular component function in the nucleus and molecular function related to nucleic acid binding and exonuclease activity acting in rRNA processing. Another two orthogroups with 20 proteins were found in all species except *C. albifundus* without functional annotation (Fig. 6b, Supplementary Tables S14, S18).

The comparative analysis between the species also identified 110 orthogroups present only in the five species of *Ceratocystis*. Some of these are shared among these *Ceratocystis* species, while others are exclusive (Fig. 6b). Out of these, 38 were common to all five species, with a total of 342 effector proteins; 13 orthogroups were found in all of the species, except *C. albifundus*; another 13 were present in *C. albifundus, C. manginecans, C. cacaofunesta,* and *C. fimbriata*; six were found in all species except *C. fimbriata*; seven in *C. cacaofunesta* and *C. manginecans*; seven in *C. cacaofunesta*, *C. manginecans* and *C. platani*; seven in *C. cacaofunesta*, *C. manginecans*, and *C. fimbriata*; four in *C. cacaofunesta* and *C. albifundus*; two in *C. albifundus, C. cacaofunesta*, and *C. fimbriata*; two in *C. manginecans* and *C. platani*; and two others present in all species except *C. cacaofunesta*. One orthogroup was found exclusively in *C. cacaofunesta*; one was present in *C. albifundus* and *C. fimbriata*; one in *C. cacaofunesta*, *C. fimbriata* and *C. platani*; and one in *C. albifundus*, *C. fimbriata* and *C. manginecans*.

**Figure 7:**
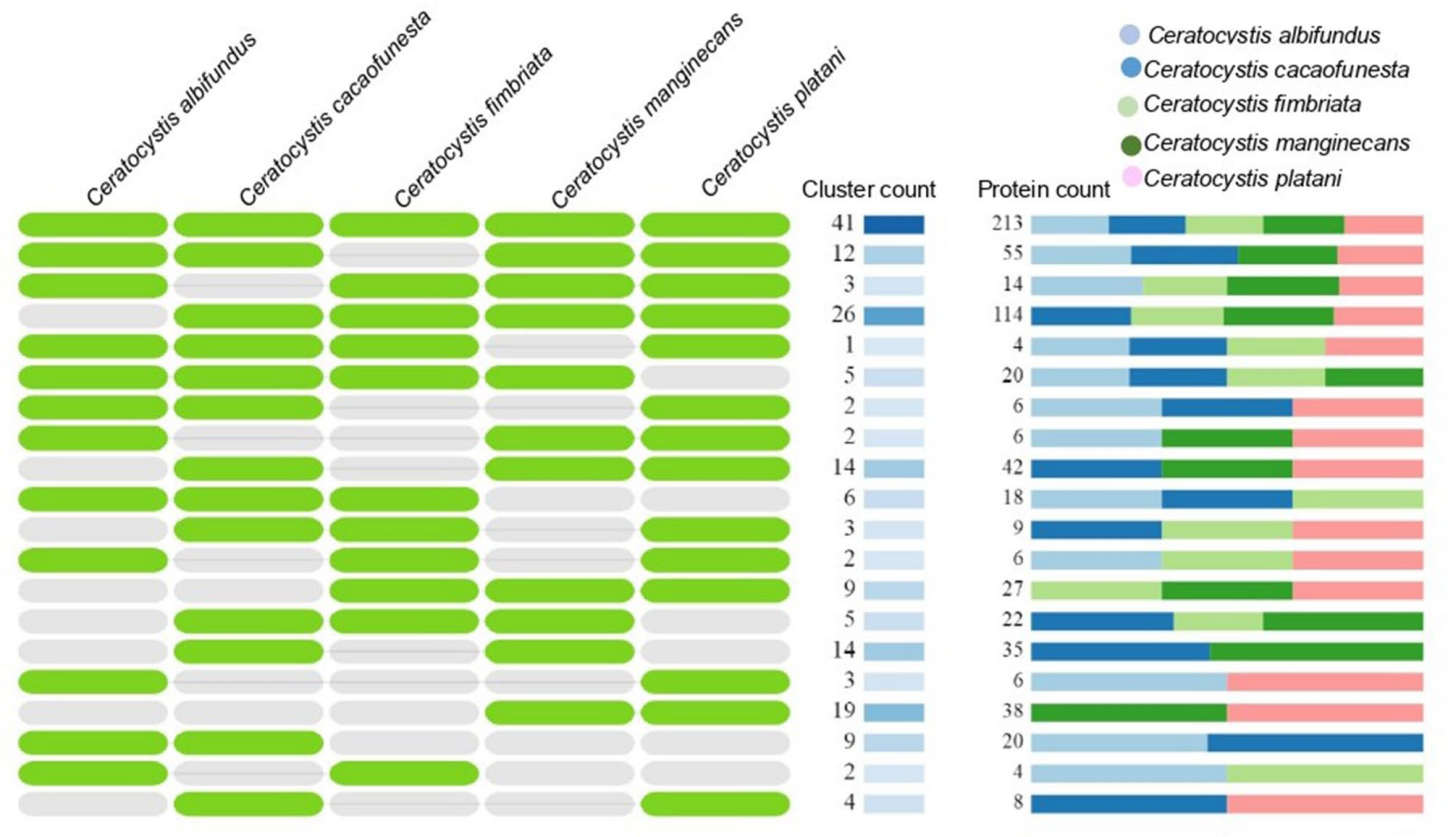
Groups of orthologous genes of five *Ceratocystis* species from OrthoVenn2 analysis. The overlay shows in the green columns the name of each *Ceratocystis* species. The cluster count column shows the number of shared orthologous groups. The protein count column indicates the number of proteins shared between orthologous groups.

The orthology analysis for the candidate effector proteins secreted by the five *Ceratocystis* species performed through Orthovenn2 shows 199 clusters, among which 161 orthogroups showed more than one copy per species and 38 groups were single-copy genes. Among the 161 orthogroups found, 41 were shared by all five *Ceratocystis* species, with 213 proteins (Fig. 7).

Among these 41 orthogroups, 12 showed functional annotation presenting similarities with proteins such as probable pectate lyase (ID: A1CYB8) and probable pectin lyase A (ID: Q4WV10). These two proteins are considered the most important in pectin depolymerization, as it breaks the internal glycosidic bonds of the highly methylated pectins. Additionally, some other similarities were found: a probable arabinan endo-1,5-alpha-L-arabinosidase A (ID: A1CLG4) also involved in pectin degradation; a secreted beta-glucosidase sun1 protein (ID: Q4WGL5), which acts on the cell surface and is involved in cell wall biosynthesis and septation and is required for normal growth and correct morphogenesis of hyphae. Other proteins such as Cell surface Cu-only superoxide dismutase ARB_03674 (ID: D4B5D4), which degrades host-derived reactive oxygen species to evade innate immune surveillance, and the probable cell wall mannoprotein PIR32 (ID: Q59PW0), being a possible cell wall structural component involved in cell wall integrity and virulence (Supplementary Tables S15, S18).

Two exclusive groups were found for *C. cacaofunesta,* with four proteins, of which three have subcellular localization in the apoplast and one in the cytoplasm. Additionally, two exclusive groups were found in *C. manginecans* with seven proteins, of which six were found with subcellular localization in the apoplast and 1 in the cytoplasm. An exclusive group for *C. platani* with two proteins that have cytoplasmic subcellular localization; however, none of these presented functional annotation in the UniProt database. The groups with single-copy genes showed 32 for *C. albifundus*, 27 for *C. manginecans*, 25 for *C. cacaofunesta*, 15 for *C. fimbriata*, and 15 for *C. platani* (Supplementary Tables S16, S17).

## Discussion

The infection by *Ceratocystis* wilt is classified as an emergent disease due to the sudden increase in the number of affected plant species and geographical areas^8^. The genomic studies based on the Ceratocystis species' pathogenicity still are few^8,44,45^, and need to be more robust. Carrying out sequencing analyses of the whole genome, as well as proteomic, transcriptomic, and bioinformatic analyses, generate the possibility of identifying genes involved in the pathogenesis, as well as the prediction of many proteins, including small proteins, generally rich in cysteine, necrosis-inducing proteins and enzymes^46,47^. A proteomic study analyzing the interaction of *T. cacao-C. cacaofunesta* showed how the presence of proteins secreted by *C. cacaofunesta* interferes with the regulation of plant proteins. During this interaction, alterations in the plant cell wall occur and a decrease in the activity of primary metabolism, directly affecting the processes of cellular respiration, photosynthesis, protein synthesis, and cell division^48^.

Previous studies on different species of *Ceratocystis* show that these genomes are small in size and have a low number of genes compared to other filamentous fungi^3,8^. Our results based on genome size comparisons performed among the five *Ceratocystis* species showed that they have very similar genomic size and gene count despite being pathogens of different hosts. The quality analysis of these genomes showed approximately 95% completeness in the five species, which is considered normal in this type of organisms that are little studied. Regarding the level of duplicated genes found in the *Ceratocystis* species, it could be because most of these pathogenic organisms have a high duplication rate to remain pathogenic and increase their genetic variability. On the other hand, future studies based on the sequencing of the complete genome would be performed to obtain a percentage of the complete integrity of these genomes.

The availability of the genomes of *C. albifundus*, *C. cacaofunesta, C. fimbriata, C. manginecans,* and *C. platani* allowed us to carry out a genetic prediction and obtain a repertoire of putative effector proteins for each of the species. The transcriptomic profile of the putative effector proteins of the species of *C. cacaofunesta* and *C. fimbriata* which revealed that most of these putative effector proteins were being expressed. Our functional annotation allowed us to obtain a set of genes involved in pathogenicity and some CAZimes.

The results from the functional annotation of putative effector proteins show that *Ceratocystis* species have similar functions. According to Molano, this could be related to their short evolutionary distance. The main functions associated with the biological processes found in the five species of *Ceratocystis* were metabolic processes of carbohydrates, lipids, and proteolysis; these processes have also been observed in *F. oxysporum,* playing an essential role in its pathogenicity^16^.

Fungi use different strategies to colonize successfully; penetration into plants is essential. In the case of necrotrophic fungi, they produce secondary metabolites and secrete different types of proteins and enzymes with which they manage to disturb host cells and induce death and the release of nutrients to facilitate the colonization of host tissues^49^. Enzymes produced by pathogens can destroy physical and chemical barriers in plants. Within the physical barriers is the cell wall, composed mainly of polysaccharides such as cellulose, hemicelluloses, and pectins. Its degradation is attributed to cell wall degrading enzymes (CWDE) related to the families of glycoside hydrolases, esterases carbohydrates, and polysaccharide lyases^50,51^.

The CAZyme analysis revealed the presence of CWDE in each of the five species of *Ceratocystis*, with an average of 12 per species, among which are some hydrolases, pectinases, oxidoreductases, and oxidases, related to the synthesis and breakdown of the plant cell wall. The presence of CAZymes in fungal pathogens is essential to achieve successful penetration and infection in their hosts^52^. Some CAZymes have already been found in different species of *Ceratocystis* but in low numbers compared to other *Sordariomycetes*, including non-pathogenic species^8^. Among the hydrolase enzymes found in the five species of *Ceratocystis*, one of the proteins was related to the degradation of cellulose of the GH11 family, specifically the endo-1,4-beta-xylanase protein showing high sequence similarity and associated with virulence in *V. dahliae* (PHI:11606), also known as the Vd424Y effector because it regulates and activates immunity by effectors and its recognition causes cell death in plants^53^. The presence of this GH11 hydrolase in our results agrees with the result obtained by Molano, who evaluated the cellulolytic activity in cultures of *C. cacaofunesta* and *C. fimbriata* presenting cellulase activity, which contributes to the degradation of the cell wall polymers of plants^8^. In other pathogens, hydrolases have also been associated with cell wall degradation, nutrient uptake, and fungal penetration of their hosts^54^.

Other proteins with sequence similarity were related to the degradation of plant pectin, pectate lyase (PL3_2), and pectin lyase (PL1_4). The presence of these possible pectinases in fungi of the genus *Ceratocystis* would directly contribute to the ability of these pathogens to attack plants, interfering in the formation of defense structures of their hosts, specifically in the formation of tyloses and in the accumulation of pectin-rich gums and gels that they are generally used against fungi that cause vascular wilt^48^. Pectinases are involved in the pathogenicity of different fungal pathogens in vascular wilt fungi such as *V. alboatrum, V. dahliae, Nectria haematococca*, and *F. oxysporum*, which have high numbers of pectinases and may be related to blockage or collapse of vascular bundles during disease development^13,52^.

In addition, the presence of a Cu-only superoxide dismutase ARB_03674 (ID: D4B5D4) cell surface protein, considered an oxidoreductase found in *Ceratocystis* species, could be involved in the removal of reactive oxygen species that are part of the defense mechanism innate of the plant. Previous studies in phytopathogenic fungi show that the presence of oxidoreductases facilitates penetration and contributes to the degradation of the host cell wall, generating components that will be used as nutrients for these fungi^55^. A study carried out on cacao plants in resistant and susceptible genotypes infected with *C. cacaofunesta* showed that this fungus can alter the defense mechanism of the plant in susceptible genotypes, causing a decrease in the production of reactive oxygen species; this could be related to the presence of the protein Cu-only superoxide dismutase ARB_03674 found in the different species of *Ceratocytis*^48^. Regarding plant lignin degradation, the main lignin-degrading organisms are white and brown rot fungi. Still, other organisms have been identified with this ability, like the pathogen *F. oxysporum,* considered a soft-rot fungus. These contain genes encoding lignin-degrading enzymes in low numbers compared to other pathogens that cause soft rot^56^. The AA11 oxidases and the enzyme peroxidase (GO:0004601), present in four species of *Ceratocystis*, are among some of the enzymes related to lignin degradation, which was found as a putative effector protein exclusive to *C. cacaofunesta.* Previous studies on *C. cacaofunesta* identified that it presents a single ligninase, relating to a possible limited ability to degrade plant lignin^8,48^.

Among the several essential virulence factors required for the colonization of plants by fungi are effector proteins, which are produced and secreted by plant pathogens. Many of these proteins are translocated to the apoplast or cytoplasm, where they alter host defense responses to enable colonization by the pathogen^57^. The genome of *C. cacaofunesta* has a wide variety of proteins with effector characteristics. Among them, we find proteins that have been studied, such as the allergen Arg and cyanovirin, which can provoke plant responses, and also proteins possibly involved in resistance to oxidative stress generated by the host^8^. Other studies show the presence of CPPs as effector proteins secreted by phytopathogenic fungi. These fungal proteins were found in some species of *Ceratocystis*, such as *C. platani, C. fimbriata,* and *C. cacaofunesta*, causing nutrient leakage and cell death in cocoa^8,58,59^. Our *in-silico* analysis shows the presence of similar sequences with the effector protein CPP present in the five proteomes with a similarity percentage of 88% and related to hypervirulence pathogenicity genes. The gene with the most significant similarity (PHI: 3167), which has functional annotation in UniProt, comes from *C. platani* and was characterized as a fungal toxin and induces cell necrosis in *Platanus acerifolia, Platanus occidentalis,* and *Platanus orientalis*^60^*.* In other fungi, such as *Botrytis cinerea* and *Sclerotinia sclerotiorum,* CPP family proteins were found to act as elicitors, inducing hypersensitive responses in plants; these responses are beneficial for the virulence of necrotrophic fungi^61^.

Most putative effector proteins found among the five *Ceratocystis* species had genes shared among the species with common functions. Others were species-specific, as Molano et al. observed, which explains their variation in pathogenicity/virulence. Of the putative effector proteins found in different species, an average of 46 show hits in the GO annotation (Fig. 3), and in our annotation performed through PHI-base, all putative effector proteins of the five species showed sequence similarity to genes associated with pathogenicity (Fig. 5). These annotation results allow us to identify potential pathogenicity genes with the similarity of amino acid sequences of other phytopathogens. Among them, we found the lethal genes that code for proteins such as Serine/threonine-protein kinase TEL1 (PHI:1224), found in four species of *Ceratocystis* except for *C. platani*. This kinase, secreted by *F. graminearum*, acts as a DNA damage sensor, activating checkpoint signaling under genotoxic stresses such as ionizing radiation (IR), ultraviolet light (UV), or paralyzed DNA replication. This protein also plays an essential role in hyphal growth and branching, conidial production, stress response, and pathogenicity of *F. oxysporum.* Within the genes associated with effectors (determinant of plant avirulence) present in different fungi, we find the Endo-1,4-beta-xylanase B protein considered an effector associated with the fungus *F. graminearum* (PHI:9746), implicated in the hydrolysis of xylan, inducing cell death and hydrogen peroxide accumulation in infected wheat leaves^62,63^. The Vd424Y effector secreted by *V. dahliae* (PHI:11606) contributes to virulence processes, triggers cell death, and induces the host's innate immunity response^53,64^.

In some of the *Ceratocystis* species, effectors were found similar to sequences related to the induction of necrotrophic cell death and possible function of the pathogen-associated molecular pattern (PAMP), among them the effectors Necrosis-inducing protein NPP1 (PHI:666) and the NLP effector protein 10 (PHI:8053) secreted by the oomycete *Phytophthora parasitica* and *Phytophthora capsici,* respectively, which moderately contribute to the virulence during the infection, generating in the plant symptoms of chlorosis and necrosis after a few days. Symptoms of chlorosis and necrosis have already been observed in different plants infected by *Ceratocystis* species. The presence of these symptoms agrees with the possible presence of these necrosis-inducing effector proteins. A study carried out in *C. cacaofunesta* and *C. fimbriata* identified that these species have two genes that code for proteins similar to NLP1 showing a concordance with our results^8^. Other studies carried out in fungi that cause vascular wilting, such as *F. oxysporum, V. dahliae,* and *V. alboatrum,* have observed the presence of these NLP family proteins in large numbers compared to other fungi, suggesting that it could be related to the wide range of hosts they have these fungi^50,65,66^.

Among the pathogenicity genes found with similarity, some reduce virulence, such as the protein Endo-1,4-beta-xylanase 11A (PHI:546) secreted by the fungus *B. cinerea,* and the protein Endo-1,4-beta-xylanase 3 (PHI:2211) secreted by *Magnaporthe oryzae,* both directly related to the hydrolysis of xylan, the second most abundant polysaccharide in the biosphere, and is necessary for plant infection and the appearance of secondary lesions^67,68^. Xylanases have also been found in fungi such as *Ustilago maydis*, which is involved in the degradation of maize plant hemicellulose, contributing to the formation of filaments on the plant surface and in the progression of hyphae within cells^69^. Our orthology results show similar protein sequences with the candidate effector candidate proteins of all five *Ceratocystis* species. Some of these proteins have already been characterized, such as the autophagy protein 27 (ID: I1RD82), a signaling effector of phosphatidylinositol 3-phosphate kinase VPS34, involved in deoxynivalenol biosynthesis. This protein is secreted by *F. graminearum* and is considered an essential determinant in its proper vegetative growth, asexual/sexual reproduction, and full virulence^70^. Genes related to proteins of the phospholipase-C family, specific to phosphatidylinositol (PI-PLC), were also found in the *C. cacaofunesta* genome. When these genes are released within the plant, they cause a destabilization of the plasmatic membrane, and when they are helped by secreted proteins that degrade the cell wall, they could contribute to the degradation of tyloses in the plant causing successful colonization for these fungi^8,48^. The protein aurofusarin biosynthesis cluster protein S (ID: I1RF63) is associated with the biosynthesis of aurofusarin, a red mycelium pigment that acts as a mycotoxin^71^.

## Conclusions

This study provides a repertoire of putative effector proteins generated from the genome of *C. cacaofunesta* and other publicly available *Ceratocystis* species, performed through in-silico gene prediction of the genome and the prediction of the secretome and effectorome*.* Additionally, it was possible to identify the expression of putative effector proteins based on the quantification of transcripts in the species *C. cacaofunesta* and *C. fimbriata*, as well as functional characteristics involved in interactions with plants, including CAZymes, hydrolases, lyases, and oxidoreductases, which show homology of sequence in all five *Ceratocystis* species*.* The identification of these putative effector proteins that have already been characterized in different phytopathogens, including species that cause vascular wilting, with functions related to cell death, necrosis, and other functions that contribute to the pathogenesis, generates a valuable resource for future studies based on the confirmation of the role of the putative effectors through wet laboratory work.

### Supplementary Information

Supplementary Table 1.

Supplementary Table 2.

Supplementary Table 3.

Supplementary Table 4.

Supplementary Table 5.

Supplementary Table 6.

Supplementary Tables.

Supplementary Table 10.

Supplementary Table 11.

Supplementary Table 12.

Supplementary Table 13.

Supplementary Tables.

## Data Availability

Supplementary Table 1 contains all accessions of the genomes, proteomes, and transcriptomes analyzed in this article, available in the NCBI and Uniprot databases. All data generated during this study are included in this article as supplementary information. Supplementary Table 2 contains the transcriptome of *C. cacaofunesta.* Supplementary Table 3 consists of the genetic predictions for *C. albifundus, C. cacaofunesta,* and *C. manginecans.* Supplementary Tables 4 and 5 contain the candidate secreted proteins and candidate effector proteins, respectively. Supplementary Table 6 contains the result of the transcriptional profile performed in *C. cacaofunesta* and *C. fimbriata.* Supplementary Tables 7, 8, and 9 have the lists of GO terms divided by category: biological processes, molecular function, and cellular component of the five species of *Ceratocystis.* Supplementary Table 10 includes the sequence alignment performed with the CPPs sequence and the Ceratocystis species' sequences. Supplementary Tables 11 and 12 contain the results of two annotations: Supplementary Table 11 of the possible CAZymes proteins possessed by the five *Ceratocystis* species, and Supplementary Table 12 has the annotation made with PHI-base to obtain potential pathogenicity genes of the five species. Supplementary Table 13 contains the 20 candidate effector proteins with the highest number of transcripts for the species of *C. cacaofunesta* and *C. fimbriata* divided into apoplastic and cytoplasmic. And finally, Supplementary Tables 14, 15, 16, 17, and 18 contain the results of the orthology analysis.
