# Supplementary material for: A repertoire of candidate effector proteins of the fungus Ceratocystis cacaofunesta
Source: Sci Rep. 2023 Sep 29;13:16368. doi: 10.1038/s41598-023-43117-7 (PMC10542334; doi:10.1038/s41598-023-43117-7)
Supplement: Supplementary file 1 — Supplementary Table 1. [file 41598_2023_43117_MOESM1_ESM.pdf]

A repertoire of candidate effector proteins of the fungus *Ceratocystis cacaofunesta*

Gabriela N. Ramos-Lizardo, Jonathan J. Mucherino-Muñoz, Eric R. G. R. Aguiar, Carlos P. Pirovani, Ronan X. Corrêa

Supplementary table S1. Identification of the genomes, proteomes and SRA of *Sordariomycetes* used in this analysis.

	Assembled genome size (Mb)	GC content (%)	Predicted proteome	Genome accession(s)	Proteome accession(s)	SRA accession(s)	Transcriptome accession(s)
Ceratocistys albifundus	26.88	48.6	7,619	GCA_000813685.1	**this paper**		
Ceratocistys cacaofunesta	30.48	48.1	7,879	GCA_002776505.1	**this paper**	SRR6217952	**this paper**
Ceratocistys fimbriata	30.16	47.6	7,266	GCA_000389695.3	UP000222788	SRR8599076	GCA_000389695.3
Ceratocistys manginecans	31.71	47.9	7,563	GCA_000712455.1	**this paper**		
Ceratocistys platani	29.18	48.2	5,622	GCA_000978885.1	UP000034841		
Verticillium albo-atrum	32.86	55.4	10,233	GCA_000150825.1	UP000008698		
Verticillium dahliae	33.9	55.6	10,530	GCA_000150675.2	UP000001611		
Fusarium graminearum	38.05	48.15	14,162	GCA_900044135.1	UP000070720		
Fusarium oxysporum	54.77	47.8	17,784	GCA_000222805.1	UP000002489		
Fusarium verticillioides	41.84	48.69	17,875	GCA_000149555.1	UP000009096		
